# Cardiovascular Disease Risk in Women with Menopause

**DOI:** 10.3390/jcm14113663

**Published:** 2025-05-23

**Authors:** María Fasero, Pluvio J. Coronado

**Affiliations:** 1 Department of Obstetrics and Gynecology, Menopause Unit, Hospital de la Zarzuela, 28023 Madrid, Spain; 2 Clínica Corofas Menopause, 13700 Tomelloso, Spain; 3 Faculty of Medicine, Universidad Francisco de Vitoria, 28223 Madrid, Spain; 4 Women’s Health Institute, Hospital Clínico San Carlos, IdISSC, 28040 Madrid, Spain; plujcoro@ucm.es; 5 School of Medicine, Universidad Complutense de Madrid, 28040 Madrid, Spain

**Keywords:** estrogens, lifestyle change, hormone replacement therapy, lipid-lowering therapy

## Abstract

**Background and objective**: Menopause is a significant physiological milestone in a woman’s life, coinciding with increased cardiovascular disease (CVD) risk due to various health-related changes. This narrative review focuses on cardiovascular health-related alterations during menopause and their implications on vascular function. **Methods**: An electronic database search was performed, drawing from sources such as PubMed and Google Scholar. Publications were included if they addressed CVD risk in peri- and postmenopausal women, and examined the impact of hormonal changes, traditional risk factors (e.g., hypertension, hyperlipidemia, diabetes), or lifestyle factors (e.g., diet, physical activity) on CVD. **Results**: Estrogen deficiency is pivotal, leading to adverse effects such as endothelial dysfunction, increased arterial stiffness, and lipid profile deterioration. Characteristics of menopause, including the age at onset, type or stage of menopause, and severity of symptoms, further modulate CVD risk. Additionally, the impact of traditional risk factors is amplified during this period. Strategies for the prevention of CVD in menopausal women are critically assessed, with a focus on lifestyle modifications, dietary interventions, and physical activity. **Conclusions**: This narrative review describes the potential benefits and risks of hormone therapy, alongside lipid-lowering therapies. Emphasis is placed on individualized risk assessment and management, highlighting the need for regular cardiovascular screenings and proactive management of risk factors.

## 1. Introduction

Although significant progress has been made in the diagnosis, prevention, and treatment of cardiovascular disease (CVD), it remains the leading cause of death globally for both men and women. However, there are differences between women and men in the presentation, diagnosis, and treatment of CVD [1,2,3]. Some CVD prevention guidelines have taken into account sex-specific conditions. However, although risk factors are considered, they do not detail the risks of each one and what to do about them. Furthermore, these guidelines consider women as a special population, so although they are revised almost annually, they have a significant baseline error [3,4,5]. This has led to underdiagnosis and undertreatment of CVD in women. Moreover, one of the key challenges in cardiovascular prevention for women is the underestimation of risk—by both healthcare providers and women themselves [6]. In fact, based on the 2012 American Heart Association (AHA) survey on women’s awareness, knowledge, and perceptions of CVD [7], along with more recent findings from the Women’s Heart Alliance survey published in 2017, only 56% of women recognize this fact [8].

While traditional CVD risk factors predominate in older age (family history, smoking, hypertension, overweight, dyslipidemia, diabetes), several specific factors may be present in middle-aged or younger women that should be taken into account when assessing the CVD risk of women at any age [4]. These risk factors may be related to pregnancy (miscarriage, premature delivery, preeclampsia, gestational hypertension, gestational diabetes), fertility (early menopause, ovarian failure, polycystic ovarian syndrome, hormone therapy [HT], endometriosis, contraceptives) or other more specific risk factors (breast cancer, autoimmune diseases, stress, depression, use and abuse of nonsteroidal anti-inflammatory drugs) [4]. Gestational diabetes and gestational hypertension are increasingly recognized as early markers for a higher risk of developing hypertension and type 2 diabetes during menopause. Women with a history of gestational diabetes and hypertension have a significantly elevated risk of developing metabolic syndrome, hypertension, and diabetes in the postmenopausal years due to the underlying insulin resistance and vascular dysfunction seen during pregnancy. These conditions act as predictors for long-term cardiovascular and metabolic health, underscoring the importance of early monitoring and intervention to reduce future risks [9,10]. On the other hand, factors like age at menarche and breastfeeding could serve as risk indicators for the early identification of women with an increased likelihood of CVD. Early menarche (at age 11 or younger) is associated with twice the likelihood of developing obesity-related hypertension in midlife. In addition, women who breastfed were less likely to be obese later in life than women who did not breastfeed [11].

CVD presents notable differences between women and men in terms of clinical presentation, outcomes, and underlying pathophysiological mechanisms, rendering it more severe in women [12]. For instance, 64% of women who die suddenly from coronary heart disease (CHD) had no prior symptoms, compared to 50% of men [13]. Additionally, following a first myocardial infarction (MI), a higher proportion of women than men die within one year (23% vs. 18%) and within five years (47% vs. 36%). Women are also more likely to develop heart failure (22% vs. 16%) or experience a stroke (7% vs. 4%) within five years of their first MI [12,14].

From the pathophysiological point of view, the incidence of ischemic heart disease in women is delayed by 10 years in men, and the incidence of MI and sudden death in women is delayed by 20 years in men [14,15]. This delay in the onset of CVD seems to be due to the cardioprotective effects of endogenous estrogens [16]. Thus, premenopausal women generally experience a protective effect against the clinical manifestations of CVD. However, following menopause, the decline in estrogen levels leads to an increased risk of cardiovascular complications. While premenopausal women have a lower risk of CVD compared to age-matched men, this risk increases after menopause [15]. According to the landmark 1976 Framingham study, among women aged 40 to 54, those who are postmenopausal experience a two- to six-fold higher incidence of CVD compared to their premenopausal counterparts [17]. It is important to keep in mind that estrogen loss can occur at any age in women, not just in old age, as there may be cases of early menopause, ovarian failure, or hysterectomy that lower estrogen levels earlier than would occur with natural menopause. The 2003 Women’s Ischemia Syndrome Evaluation (WISE) study found that young women with estrogen deficiency face a sevenfold higher risk of coronary artery disease compared to those without this deficiency [18].

This narrative review aims to synthesize the relationship between menopause and CVD in women, what characteristics of menopause are responsible for the increased CVD risk, and what strategies can be used to prevent CVD in menopausal women.

## 2. Methods

This review focuses on providing a comprehensive overview rather than a meta-analytic summary. An electronic database search was performed, drawing from sources such as PubMed and Google Scholar. Search terms combined Medical Subject Headings (MeSH) and free-text keywords, such as “menopause”, “cardiovascular disease”, “risk factors”, “hormonal changes”, and “postmenopausal women”. Publications were included if they addressed CVD risk in peri- and postmenopausal women, and examined the impact of hormonal changes, traditional risk factors (e.g., hypertension, hyperlipidemia, diabetes), or lifestyle factors (e.g., diet, physical activity) on CVD. Articles were excluded if they focused solely on men or premenopausal women and did not address CVD risk explicitly.

## 3. Menopause as a Risk Factor for CVD

Menopause is defined as a permanent cessation of menstruation, which is confirmed retrospectively after 12 consecutive months of amenorrhea without any underlying pathological causes [19]. Menopause occurs as a result of the cessation of estrogen production, driven by the stimulation of follicle-stimulating hormone (FSH) and luteinizing hormone (LH) due to ovarian follicular atresia. Studies have reported a decline in estradiol levels two years prior to the final menstrual period, with an increase in FSH levels observed up to six years before this time point [20].

The menopausal transition begins 5 to 10 years before the last menstrual period. Based on the degree of menstrual cycle variability, women can be classified into the early transition stage (defined by a persistent difference of at least 7 days in consecutive menstrual cycle lengths) or the late transition stage (characterized by at least one instance of amenorrhea lasting 60 days or more). Perimenopause refers to the first year following the last menstrual period. It marks a period of increased variability in the length and consistency of the menstrual cycle, as well as increased frequency of menopausal symptoms (hot flashes, night sweats, mood changes, sleep, and cognitive disturbances, as well as genitourinary and sexual function changes) [21]. During this period, estrogen and progesterone levels begin to fluctuate. Postmenopause refers to the period following a woman’s last menstrual period, during which estrogen and FSH levels stabilize [19].

Natural menopause typically occurs around the age of 50 [18]. However, it is classified as premature if it occurs between the ages of 40 and 45. About 10% of women experience menopause before the age of 45 [22]. Factors influencing natural menopause timing include age, race/ethnicity, weight and body mass, physical activity, diet, alcohol consumption, smoking, reproductive history factors, and genetics [15].

### 3.1. Cardiovascular Health-Related Changes During Menopause

Throughout the menopausal process, estrogen levels are decreasing, while important cardiometabolic changes occur that go beyond the woman’s chronological age: increased levels of total cholesterol, low-density lipoprotein (LDL)-cholesterol, triglycerides and apolipoprotein B; decreased high-density lipoprotein (HDL)-cholesterol; increased visceral fat (which doubles or triples during this phase of life); and progression and increased severity of metabolic syndrome (especially during the late premenopause and perimenopause) (Table 1) [23,24].

In contrast, menopause, by itself, was not found to be independently associated with increases in blood pressure, insulin, or glucose levels beyond those attributed to aging [25,26], and changes in weight and body mass index observed over time in women do not appear to be related to menopause (both in premenopausal and postmenopausal women) [27]. That is, changes in weight were more strongly associated with chronological aging than with reproductive aging. All this means that menopause has been considered by the AHA in 2020 as an independent risk factor for CVD and accelerated progression of atherosclerosis in women [15].

Existing studies analyze the differences between premenopausal and postmenopausal women. One study found that premenopausal women have a statistically lower risk of CVD compared to postmenopausal women (OR = 0.338, *p* < 0.001) [28].

While the timing of menopause onset is believed to influence CVD risk, the relationship may be bidirectional. The Framingham study suggests that higher levels of total cholesterol, systolic and diastolic blood pressure, and other CVD risk factors before menopause are linked to earlier menopause, independent of smoking [29]. Similarly, experiencing a first episode of CVD before the age of 35 has been linked to a doubling of the risk for early menopause, while a first episode occurring after 35 is associated with menopause at around age 51 [30]. Thus, poorer premenopausal cardiovascular health may influence the onset of menopause [30].

### 3.2. Characteristics of Menopause About the Risk of CVD

**Figure 1:**
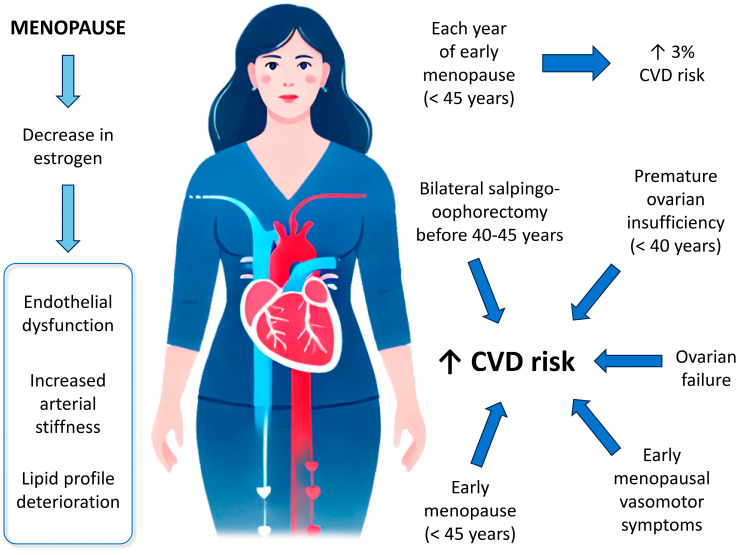
Menopause and risk of CVD. ↑: increase; CVD: cardiovascular disease.

Various menopause characteristics have been assessed in relation to CVD risk, including the age of onset, type and stage of menopause, endogenous estradiol levels, and menopause-related symptoms. Several of these factors have been found to be associated with CVD risk [31]. Figure 1 shows a summary of all of them.

#### 3.2.1. Age of Onset of Menopause

Multiple meta-analyses have found that women with early-onset menopause (before age 45) face a significant higher risk of both overall and fatal CHD, as well as an increased risk of heart failure, compared to those experience menopause at age 45 or older [32,33]. Additionally, each year of early menopause is associated with a 3% increase CVD risk [34].

#### 3.2.2. Type or Cause of Menopause

The risk of CHD is higher in women who undergo bilateral salpingo-oophorectomy (BSO) without estrogen therapy compared to those who experience natural menopause [35]. While there is little to no association between BSO and CVD risk when BSO occurs near the time of natural menopause, the risk is significantly elevated when BSO occurs at a younger age (before 40–45 years) [36]. Similarly, the risk of composite CVD in women with BSO before the age of 40 is comparable to that in women with premature natural menopause (before 40) [37].

The highest CVD risk is observed in women with ovarian failure, early menopause (ages 40–44), and relatively early menopause (ages 45–49) [34]. Women with premature ovarian failure tend to have a shorter life expectancy compared to those with later menopause, primarily due to an increased risk of CVD and osteoporosis [32,38,39].

#### 3.2.3. Stage of Menopause

A cross-sectional study involving women aged 44 to 56 at various menopausal stages found that both systolic and diastolic blood pressure were significantly higher only in the late menopause compared to early menopause [40]. In contrast, the SWAN analysis reported that levels of total cholesterol, HDL-cholesterol, LDL-cholesterol, triglycerides, and lipoprotein(a) peaked during late perimenopause and early postmenopause compared to the premenopausal phase [41]. Additionally, the SWAN analysis indicated that structural remodeling of the carotid artery was most pronounced during late perimenopause compared to both premenopause and early perimenopause [42].

#### 3.2.4. Endogenous Estrogens Levels

The decline in endogenous estradiol levels during menopause has also been linked to alterations in CVD risk factors. However, the supporting evidence is limited because it is focused mainly on postmenopausal women [20]. In contrast, studies relating estradiol levels to subclinical measures of atherosclerosis have been more consistent. Among late perimenopausal and postmenopausal women, elevated estradiol levels were associated with smaller carotid inter-adventitial diameter (indicating less carotid remodeling), and with enhanced flow-mediated dilation of the brachial dilatation (i.e., better endothelial function) [43].

#### 3.2.5. Vasomotor Symptoms

Menopausal vasomotor symptoms have been associated with unfavorable lipid profile, insulin resistance, and a heightened risk of hypertension [20,21]. A meta-analysis comparing women with and without menopausal symptoms found that vasomotor and other menopausal symptoms were generally linked to an increased risk of CHD, stroke, or CVD [44]. Additionally, the SWAN study reported that women experiencing hot flashes exhibited reduced flow-mediated dilation and increased aortic calcification compared to those without hot flashes, independent of traditional CVD risk factors and estradiol level [45]. In a further study, women with hot flashes at both baseline and 2-year follow-up had greater carotid intima–media thickness than those who did not report hot flashes, especially among overweight or obese women [46]. Some studies have found that CVD risk may depend on the timing or duration of vasomotor symptoms. Another analysis from the SWAN study found that women who experienced vasomotor symptoms early in menopause had greater mean and maximal carotid intima–media thickness compared to those with consistency low and stable frequencies of these symptoms [47].

#### 3.2.6. Other Symptoms of Menopause

Estrogen and progesterone changes during menopause have unique effects related to sleep. The overall decline in estrogen can contribute to hot flashes, night sweats, mood disturbances, and symptoms of anxiety and depression, all of which may result to frequent nighttime awakenings, disrupted sleep, poor sleep quality, and insomnia. Simultaneously, the reduction in progesterone (a hormone with natural sedative and calming effects) can further impair sleep. Additionally, some studies have associated the decline in these hormones during perimenopause with higher rates of snoring and obstructive sleep apnea [48]. According to several investigations performed in women at different stages of menopause, significant associations were seen between poorer sleep quality and increased risk of metabolic syndrome and increased carotid atherosclerosis. These relationships remained significant even after adjusting for CVD risk factors, hot flashes, and estradiol levels [49,50]. Moreover, poor sleep quality has been independently linked to a higher risk of aortic calcification and increased arterial stiffness in perimenopausal women, but not in those who are premenopausal [51,52].

Depressive symptoms that appear during menopause have also been associated with an increased risk of CVD. According to the SWAN study performed in healthy women aged 46–59 years with a follow-up of 5 years, having ≥3 episodes of depression versus not having them was associated with greater coronary artery calcification [53]. It has also been shown that in postmenopausal women without a prior history of CVD, depression independently predicted CVD-related death and all-cause mortality over an average period of 4.1 years, even after controlling for demographic factors and established CVD risk factors [54].

In regression analyses examining palpitations, chronic cardiovascular disease, hot flushes, night sweats, and depressed mood were identified as significant predictors. These findings suggest a link between physical health (such as pulmonary and CVD), vasomotor symptoms (hot flushes and night sweats), psychological distress, and lifestyle factors (lack of physical activity, use of oral contraceptives, alcohol consumption, and smoking) in experiencing palpitations, particularly during midlife and menopause [55].

#### 3.2.7. Novel Biomarkers for Cardiovascular Risk Prediction in Postmenopausal Women

Recent research has identified several novel biomarkers that may improve cardiovascular risk prediction in postmenopausal women beyond traditional risk factors. Among these, high-sensitivity C-reactive protein (hs-CRP) and lipoprotein(a) have emerged as strong indicators of vascular inflammation and genetic predisposition to atherosclerosis, respectively [56,57,58,59,60]. Additionally, adiponectin, a hormone involved in glucose regulation and fatty acid oxidation, shows an inverse relationship with CVD risk and may be particularly relevant in the context of menopause-related metabolic changes [61]. New studies have also highlighted the role of microRNAs—small non-coding RNAs involved in gene regulation—as promising biomarkers that reflect endothelial dysfunction and systemic inflammation in menopausal women [62].

Emerging research suggests that the gut microbiome’s composition and its metabolites, such as trimethylamine-N-oxide (TMAO), could offer valuable insights. Studies have linked dysbiosis and elevated TMAO levels to increased cardiovascular events in various populations [63].

Furthermore, arterial stiffness measurements using pulse wave velocity and carotid intima–media thickness have gained traction as non-invasive, predictive imaging markers [64]. These advancements underscore the importance of a personalized, sex-specific approach to CVD prevention in menopausal populations and may eventually reshape clinical risk assessment models.

## 4. Strategies for the Prevention of CVD During Menopause

To reduce the risk of CVD in menopausal women, several strategies can be followed that can be complementary to each other.

### 4.1. Hormone Therapy

The increased risk of CVD after menopause offers an opportunity to extend endogenous estrogen cardioprotection in postmenopausal women, with HT serving as a primary, sex-specific strategy for preventing CVD and reducing all-cause mortality. That is, receiving estrogen HT may help restore cardioprotective benefits while treating menopause symptoms. However, although observational studies of HT after menopause have shown promising results in reducing CVD risk, larger randomized controlled trials have failed to demonstrate any benefit in primary or secondary prevention [65]. HT is not approved by the Food and Drug Administration (FDA) for primary prevention of CVD and is only indicated for the treatment of menopausal symptoms.

Overall, HT does not appear to have a significant effect on the risk of MI. The continuous combination of estrogens (e.g., estradiol) and progestogens (e.g., norethisterone acetate, medroxyprogesterone, or norgestrel) appears to increase the risk, whereas transdermal, vaginal estrogen reduces the risk [66]. The use of conjugated equine estrogens (CEE) combined with medroxyprogesterone acetate (MPA) has also been associated with an elevated risk of MI and an increase in ischemic heart disease events compared to placebo, although this effect was not significant over the long term [65,67,68].

It appears that the timing of HT initiation could play an important role in reducing CVD risk. If it is started at the right time (especially under age 60 and at or near menopause), HT could reduce death from heart disease and death from any cause [69]. That timing is critical, as this is when HT protects blood vessels and reduces atherosclerosis. Starting HT too late can accelerate these complications and increase the risk of CVD. The importance of the initiation of HT seems to lie in the “healthy endothelium hypothesis” which proposes that HT has beneficial effects when started early on healthy endothelium, but may have harmful effects in the presence of established atherosclerotic plaques [70].

Interestingly, while observational studies showed a CVD risk reduction of 30–50% in women using HT [71,72], randomized studies did not show any benefit from this therapy. This inconsistency seems to stem from variations in the characteristics of the female populations studied [73]. While observational studies primarily involved typical HT users—relatively young women (aged 30–55), within two years of menopause onset, generally lean, and experiencing symptoms such as hot flashes—randomized controlled trials included participants who were significantly older (average age over 63 years) and, on average, more than 10 years postmenopause. However, consistent with findings from observational studies, two meta-analyses of randomized controlled trials demonstrated that HT significantly reduced all-cause mortality by 39% and CHD by 32% when started in women under 60 years of age and/or within 10 years of menopause. In contrast, no significant effect on all-cause mortality or CHD was observed when HT was initiated in women over 60 or more than 10 years postmenopause [74,75].

Compared to other therapies for primary CVD prevention, HT demonstrates an excellent risk profile, particularly when initiated in women under 60 years of age or within 10 years of menopause. For instance, the risk of breast cancer associated with statin use is comparable to that of continuous combined CEE+MPA therapy [65]. Moreover, while statins are linked to an increased risk of new-onset diabetes mellitus, exceeding what is considered a minimal risk [76], HT has been shown to improve insulin sensitivity, enhance glucose tolerance, and lower the incidence of new-onset diabetes by 20% to 30% [77]. Recent findings also suggest a 29% increased risk of gastroesophageal reflux disease associated with HT use [78]. Nevertheless, HT is contraindicated in certain populations, including women with a history of thromboembolism, uncontrolled hypertension, or breast or uterine cancer [79].

Finally, the cardiovascular health effects of discontinuing HT remain unclear, with studies yielding mixed results. Findings from the Women’s Health Initiative (WHI) indicated that 8.2 years after stopping CEE+MPA therapy and 6.6 years after discontinuing CEE alone, there were no significant differences in the incidence of CHD, stroke, pulmonary embolism, and all-cause mortality [51]. However, a Finnish study showed a more than 2-fold increase in the risk of CVD or stroke mortality one year after the end of treatment [80]. It is important to recognize that the two studies differed in both design and study populations, and the Finnish study did not specify the reasons for discontinuing HT or whether any risk factors were present during its use.

Table 2 shows a summary of the pros and cons of HT for CVD prevention in postmenopausal women.

HT in women with pre-existing cardiovascular conditions needs careful consideration. For women with a history of MI, coronary artery disease, or spontaneous coronary artery dissection, HT is generally not recommended due to the increased risk of heart events. In women with heart failure, HT may worsen symptoms due to potential fluid retention. HT is also contraindicated in women with a history of deep vein thrombosis, pulmonary embolism, pulmonary hypertension, or cardiomyopathies, as it can increase the risk of blood clots or exacerbate heart failure symptoms. For these women, the risks of HT may outweigh the benefits. Non-hormonal alternatives, such as lifestyle changes, statins, or selective estrogen receptor modulators (SERMs), are often considered safer for managing menopausal symptoms and cardiovascular health [67,79,81,82].

### 4.2. Lifestyle Changes

Achieving ideal cardiovascular health in women with menopause also requires lifestyle changes. These changes include smoking cessation, exercise, weight loss, blood pressure and lipid levels control, and following a diet such as the Mediterranean diet or Dietary Approaches to Stop Hypertension (DASH). Although there is strong evidence to support these changes to reduce the burden of CVD, there are few studies that have been performed specifically in menopausal women.

As we stated before, peri- and postmenopausal women are more likely to have more sleep disturbances than those in premenopause, and women with poor sleep have a higher CVD risk [49,52]. Although the main recommendation is to obtain more and better sleep, it can be a difficult task, especially if other symptoms like hot flashes and night sweats occur at night. However, adopting good sleep hygiene practices may promote better rest. These include maintaining a consistent sleep schedule, engaging in physical activity during the day, and incorporating calming activities before bedtime to facilitate falling asleep. As alternatives, some supplements may be considered. Phytoestrogens (i.e., isoflavone supplements) appear to reduce the frequencies of hot flushes [83], and saffron extract could reduce the psychological symptoms of perimenopause [84]. All these can help in falling asleep.

Several randomized studies and meta-analyses have investigated the potential benefits of dietary and physical-activity interventions in middle-aged women, including those at various stages of menopause. Notably, the Women’s Healthy Lifestyle Project (WHLP) was specifically developed to evaluate the impact of a combined diet and exercise program in premenopausal women [85]. In this trial, healthy premenopausal women aged 44 to 50 were randomly assigned to either an assessment-only control group or a five-year cognitive–behavioral intervention that promoted a hypocaloric diet low in saturated fat and cholesterol, along with moderate increased in leisure-time physical activity. After 54 months, the intervention proved effective in mitigating the increase in LDL-cholesterol, preventing weight gain during the transition from premenopause to peri- or postmenopause, and reducing triglycerides, systolic and diastolic blood pressure, blood glucose levels, and insulin [85]. Moreover, the Nurses’ Health Study offered compelling evidence supporting the positive impact of maintaining a healthy lifestyle (encompassing a balanced diet, regular physical activity, and smoking cessation) on lowering the risk of CHD in women [86]. According to a recent systematic review and meta-analysis, resistance training helps to reduce total LDL-cholesterol and triglycerides in postmenopausal women [87].

### 4.3. Lipid-Lowering Therapy in Women

To improve the lipid profile, the first recommendation is to modify lifestyle (weight loss, exercise, smoking cessation, healthy diet). However, in necessary cases, lipid-lowering therapy may be required. Most of the studies that have evaluated these therapies have focused on hydroxymethylglutaryl-CoA (HMG-CoA) reductase inhibitors (i.e., statins). Although the evidence on the use of statins in women is limited and controversial, the JUPITER trial examined a cohort of women with baseline LDL-cholesterol levels below 130 mg/dL and hs-CRP levels above 2 mg/dL [88]. The findings showed that rosuvastatin significantly reduced cardiovascular events in women without prior CVD, demonstrating a relative risk reduction comparable to that observed in men, a result further supported by a meta-analysis of statin trials focused on primary prevention [88].

While statins remain the primary therapy for lowering LDL-cholesterol, additional or alternative treatments may be appropriate for optimizing lipid profiles in cases of insufficient LDL-cholesterol control or statin intolerance. These alternatives include bile acid sequestrants, cholesterol absorption inhibitors (ezetimibe), and PCSK9 (proprotein convertase subtilisin/kexin type 9) inhibitors [89,90]. A major limitation of these lipid-lowering therapies is that current CVD prevention guidelines do not offer tailored recommendations for women, instead categorizing them within broader special population groups [3].

## 5. Conclusions

CVD risk significantly increases in menopausal women due to hormonal and metabolic changes. Emphasizing individualized risk assessment and management is crucial, incorporating regular cardiovascular screenings and proactive management of risk factors such as hypertension, dyslipidemia, and obesity. Tailored lifestyle interventions and, where appropriate, therapeutic strategies can mitigate these risks, improving cardiovascular outcomes for postmenopausal women. Clinicians and researchers must be understanding of the complex relationship between menopause and cardiovascular health to foster improved cardiovascular outcomes for menopausal women.

## Figures and Tables

**Table 1:** Cardiovascular health-related changes that occur with menopause beyond chronological age [15].

Change in lipids	↑ Total cholesterol ↑ LDL-cholesterol ↑ Apolipoprotein B ↓ HDL-cholesterol
Metabolic changes	↑ Metabolic syndrome
Change in vessels	↑ Carotid atherosclerosis ↑ Carotid adventitial diameter ↑ Carotid intima–media thickness ↑ Arterial stiffness
Body changes	↑ Fat mass ↓ Lean mass ↑ Ectopic fat deposition (especially in heart and liver)

↑: increase; ↓ decrease; HDL: high density lipoprotein; LDL: low density lipoprotein.

**Table 2:** Pros and cons of HT for CVD prevention in postmenopausal women.

Pros of HT	Cons of HT
Reduction in menopausal symptoms: HT alleviates common symptoms such as hot flashes, night sweats, and vaginal dryness.	Increased risk of breast cancer: Long-term use of HT, especially combined estrogen and progestin, is associated with a higher risk of breast cancer.
Potential improvement in lipid profiles: HT may lead to improved cholesterol levels, such as increased HDL-cholesterol and decreased LDL-cholesterol.	Increased risk of blood clots: HT can raise the risk of deep vein thrombosis and pulmonary embolism, especially in older women or those with other risk factors.
Possible reduction in coronary artery disease risk: Early initiation of HT (around the time of menopause) may reduce the risk of heart disease, particularly in younger women.	Increased risk of stroke: Some forms of HT, especially oral estrogen, have been linked to an elevated risk of ischemic stroke.
Bone health benefits: HT helps in maintaining bone density, reducing the risk of osteoporosis and fractures.	Potential increased risk of cardiovascular events: Evidence suggests HT may not significantly reduce cardiovascular risk and could even increase the risk in some women, particularly those who start HT later after menopause.
Improved endothelial function: HT has been shown to improve endothelial function, which is important for cardiovascular health.	Mood swings and mental health issues: Some women experience mood swings, anxiety, or depression as side effects of HT.
Possible protective effect on cognitive function: Early initiation of HT may help in preserving cognitive function and reduce the risk of dementia in some women.	Not suitable for all women: HT is contraindicated in women with a history of certain cancers (e.g., breast or endometrial), liver disease, or unexplained vaginal bleeding.

HDL: high density lipoprotein; HT: hormone therapy; LDL: low density lipoprotein.

## Abbreviations

The following abbreviations are used in this manuscript:

AHA	American Heart Association
BSO	Bilateral salpingo-oophorectomy
CEE	Conjugated equine estrogens
CHD	Coronary heart disease
CVD	Cardiovascular disease
DASH	Dietary Approaches to Stop Hypertension
FDA	Food and Drug Administration
FSH	Follicle-stimulating hormone
HDL	High-density lipoprotein
HMG-CoA	Hydroxymethylglutaryl-CoA
hs-CRP	high-sensitivity C-reactive protein
HT	Hormone therapy
LDL	Low-density lipoprotein
LH	Luteinizing hormone
MeSH	Medical Subject Headings
MI	Myocardial infarction
MPA	Medroxyprogesterone acetate
PCSK9	Proprotein convertase subtilisin/kexin type 9
WHI	Women’s health initiative
WHLP	Women’s Healthy Lifestyle Project
WISE	Women’s Ischemia Syndrome Evaluation
